# Potential anti-obesity effects of two-graded doses of Iraqi *Hibiscus tiliaceus* leaves extract, alone and in combination with orlistat, on high-fat diet-induced obesity in male rats

**DOI:** 10.25122/jml-2023-0140

**Published:** 2023-09

**Authors:** Saba Khaldoon Mohammed, Shihab Hattab Mutlag

**Affiliations:** 1 Department of Pharmacology and Toxicology, College of Pharmacy, University of Baghdad, Baghdad, Iraq

**Keywords:** alanine, aspartate aminotransferases, transaminase, hibiscus, orlista

## Abstract

Obesity is a world health concern and a serious risk factor for several chronic diseases. *Hibiscus tiliaceus* is a plant with reported anti-obesity properties. However, the preclinical anti-obesity effect of ethanolic extract of Iraqi *Hibiscus tiliaceus* has not been studied yet. This study aimed to evaluate the preclinical anti-obesity properties of Iraqi *Hibiscus tiliaceus* extract, alone or in combination with orlistat, on high-fat diet-induced obesity in male rats. Male rats were divided into five groups: control, induction, ethanolic extract of Iraqi *Hibiscus tiliaceus* (250 mg/kg and 500 mg/kg), orlistat (Xenical) alone (10 mg/kg), and a combination of the extract (250 mg/kg) with Xenical. The rats were fed a high-fat diet to induce obesity, and treatments were given orally for 8 weeks. Body weight, food intake, serum lipid profile, and liver enzymes were measured. Administration of ethanolic extract of Iraqi *Hibiscus tiliaceus* (250 mg/kg and 500 mg/kg), Xenical alone (10 mg/kg), and combination with the extract (250 mg/kg) for 8 weeks significantly reduced body weight, food intake, serum triglycerides, total cholesterol, low-density lipoprotein cholesterol, and liver enzymes (aspartate transaminase and alanine transaminase) when compared to the induction group. The ethanolic extract of Iraqi *Hibiscus tiliaceus* showed anti-obesity effects and could be a potential therapeutic agent in managing obesity. However, further studies are needed to evaluate its clinical efficacy and safety.

## INTRODUCTION

Obesity is a growing global health concern associated with various diseases and complications, such as hypertension and type 2 diabetes mellitus. Due to the risks and potential unsustainability of synthetic anti-obesity drugs, there is a growing interest in exploring natural alternatives. One such alternative is using extracts from *Hibiscus tiliaceus* leaves, which have been used for medicinal purposes for centuries and may offer a safer and more sustainable approach to managing obesity [1, 2].

The prevalence of obesity is a matter of global concern, significantly impacting public health. This condition is associated with an increased risk of developing co-morbidities, including dyslipidemias, cardiovascular disease, and type 2 diabetes, throughout an individual's lifespan. Various approaches are available for weight loss, ranging from lifestyle modifications such as dietary changes and exercise to medication and surgical interventions. While lifestyle changes can lead to short-term success in managing obesity, they often fail to achieve long-term results. Therefore, medication may be necessary as a complementary strategy to lifestyle modifications for individuals affected by obesity [3].

While lifestyle and behavioral changes are crucial in managing obesity, some individuals do not respond well to these interventions or struggle to maintain initial weight loss results. For such individuals, new medications provide a potential treatment option [3, 4].

The National Institutes of Health recommends using anti-obesity medications for individuals with a body mass index (BMI) of 30 or 27 kg/m^2^ and dyslipidemia, hypertension, or diabetes [5]. The US Food and Drug Administration (FDA) has recently approved several anti-obesity drugs for extended use beyond 12 weeks, including liraglutide, phentermine/topiramate controlled-release (CR), naltrexone extended-release (ER)/bupropion ER, and orlistat [6].

Orlistat and other anti-obesity medications have been registered for prolonged use in several countries. Its mechanism of action involves the inhibition of pancreatic, gastric, and intestinal lipases, thereby impeding the breakdown of triglycerides into fatty acids and preventing their absorption in the intestines. This process leads to weight loss by limiting the absorption of fatty acids in the intestines [7-10].

In contrast, natural extracts, such as *Hibiscus tiliaceus* leaves, offer a potentially safer and more sustainable alternative to synthetic anti-obesity drugs. Synthetic drugs can carry harmful side effects and pose sustainability challenges in production. In contrast, natural extracts like *Hibiscus tiliaceus* leaves have a long history of medicinal use and may have fewer adverse effects. Furthermore, the use of natural extracts promotes sustainable agricultural practices. One study indicated that the extract from *Hibiscus tiliaceus* leaves could be a safer and more sustainable alternative to synthetic drugs for treating obesity [11].

*Hibiscus tiliaceus* L is a plant belonging to the *Malvaceae* family. The plant is a mangrove flourishing in tropical Asia and abundant in forests. The leaves of this plant were used in traditional medicine to cure fevers, coughs, ulcers, wounds, and different skin ailments [12].

*H. tiliaceus* was employed as a febrifuge, laxative, resolvent, and emollient in Indian medicine. Its fruit juice has been applied to the skin to treat weakness [13-15]. This research investigated the possible anti-obesity effects of two-graded doses of Iraqi *Hibiscus tiliaceus* leaves extract, alone and in combination with orlistat, on high-fat diet-induced obesity in male rats[16-19]. The research aims to present arguments in favor of *Hibiscus tiliaceus* leaves extract and orlistat combination in reducing body weight and fat mass, improving lipid profile and glucose metabolism, and promoting sustainable agricultural practices.

## MATERIAL AND METHODS

### Plant collection

*Hibiscus tiliaceus* was bought from Baghdad, and the leaves were collected from September 2022 to January 2023 and authenticated at the Al Razi Centre for Alternative Medicine.

### Orlistat preparation

Orlistat was purchased from MACKLIN. The quantity required was freshly prepared by dissolving it in animal ghee.

### Hot continuous extraction or Soxhlet extraction

The leaves of *Hibiscus tiliaceus* were dried and ground into coarse particles. The powdered material underwent a defatting process by soaking it in n-hexane for 72 hours [20]. Subsequently, 60 grams of the powdered material were placed in a thimble and extracted with ethanol using a Soxhlet extraction apparatus. The extraction process was conducted for approximately 20 hours to ensure the extraction of all possible ingredients [21]. The resulting extract was dried using a rotary evaporator at 40°C, yielding a semi-solid extract. The semi-solid extract was further dried until a solid extract was obtained, which was then ready for use.

### Experiment protocol

Five- to six-week-old Wistar rats weighing between 90-140 g, obtained from the Animal House at the College of Pharmacy, University of Baghdad, were used in this study. The rats were housed in seven cages, with eight rats in each cage for Groups II, III, and IV and four rats in each cage for Groups I and V. The animals were kept in a controlled environment with a standard chow diet and unrestricted access to water. The housing facility maintained a 12-hour light-dark cycle and a constant temperature of 25ºC±1.

The rats were randomly assigned to five groups, each consisting of eight animals. The groups were organized as follows:

**Group I:** Rats were fed a high-fat diet for 8 weeks to induce obesity and served as the control group.

**Group II:** Rats were fed a high-fat diet for 8 weeks and received 250 mg/kg [22] of *Hibiscus tiliaceus* leaf extract.

**Group III:** Rats were fed a high-fat diet for 8 weeks and received 500 mg/kg [22] of *Hibiscus tiliaceus* leaf extract.

**Group IV:** Rats were fed a high-fat diet for 8 weeks and received 250 mg/kg of *Hibiscus tiliaceus* leaf extract and 10 mg/kg [23] of orlistat.

**Group V:** Rats were fed a high-fat diet for 8 weeks and received 10 mg/kg of orlistat.

The weight of the animals in all five groups was measured weekly, starting from the beginning of the experiment.

### Induction of obesity

Rats were fed food that contained 70% of normal chow mixed with 30% of animal ghee to induce obesity in all groups.

### Serum preparation

After 8 weeks, all rats were euthanized by diethyl ether [24]. Euthanasia was performed by cutting the carotid artery, followed by cervical dislocation. The blood was collected from the carotid artery into a gel tube and coagulated for 15-30 minutes [26]. After coagulation, it was centrifuged for twenty minutes at 3000 rpm to obtain serum. The serum was collected using a micropipette, transferred into Eppendorf tubes, and then stored at -20°C until further analysis. The serum was used to estimate the lipid profile, including HDL, cholesterol, and triglycerides, using a biochemical method.

### Statistical analysis

Statistical analyses were performed using the Statistical Package for the Social Sciences (SPSS) software. Group comparisons were conducted using a one-way analysis of variance (ANOVA) to determine statistically significant differences among the experimental groups. A significance level of p<0.05 was used to determine statistical significance. The results are presented as mean ± standard deviation.

## RESULTS

### Effect of *Hibiscus tiliaceus* leaves extract on body weight

**Figure 1:**
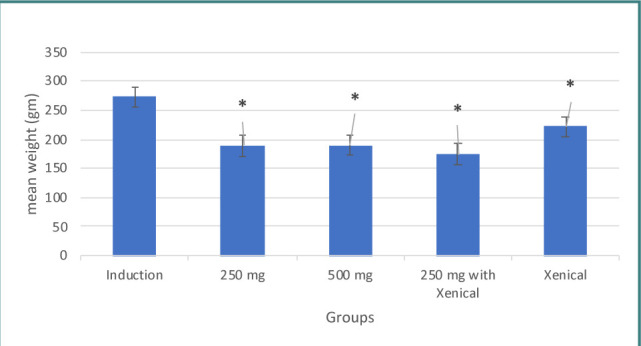
Effect of treatment on body weight

Both doses of *Hibiscus tiliaceus* leaves extract, 250 mg/kg and 500 mg/kg, demonstrated a significant anti-obesity effect compared to the induction group, as shown in Table 1 and Figure 1.

**Table 1:** Effect of treatment on body weight

Groups	Mean weight (gm)
Induction	272.36±30.557
*Hibiscus tiliaceus* 250 mg	189.03±18.963^*^
*Hibiscus tiliaceus* 500 mg	189.58±17.447^*^
*Hibiscus tiliaceus* 250+Xenical	174.44±21.471^*^
Xenical	221.39±41.117^*^

Data are presented as mean±standard deviation (*) significant difference (p<0.05) when compared to the induction group

### Effect of Xenical on bodyweight

Xenical showed a significant anti-obesity effect compared to the induction group (Table 1 and Figure 1).

### Effect of combining herbal extract and Xenical on bodyweight

The combination of herbal extract and Xenical showed significant anti-obesity effects compared to the induction group, as shown in Table 1 and Figure 1.

### Effect of *Hibiscus tiliaceus* leaves extract on lipid profile

**Figure 2:**
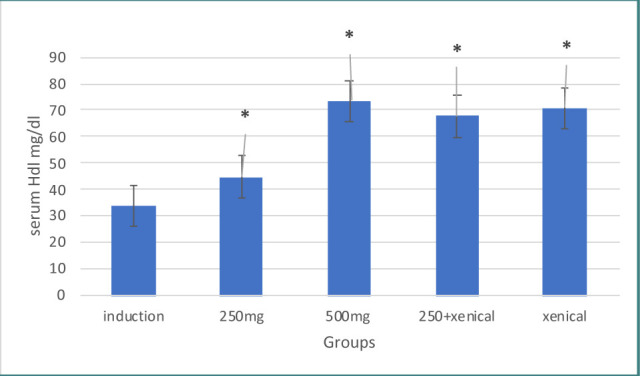
Effect of treatment on HDL

**Figure 3:**
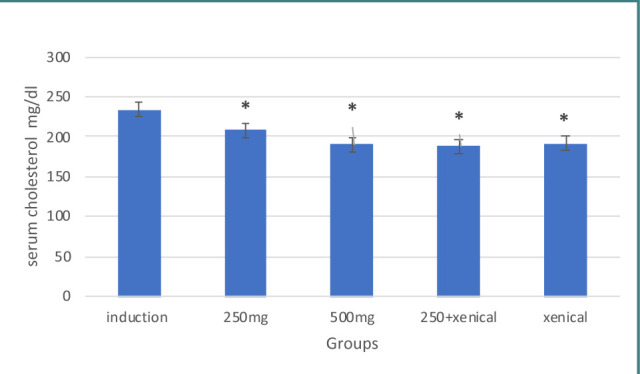
Effect of treatment on cholesterol

**Figure 4:**
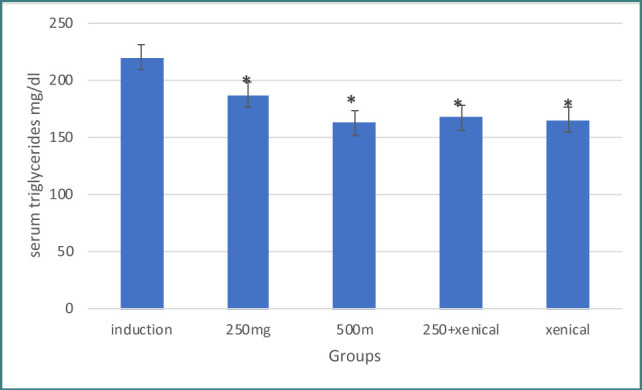
Effect of treatment on triglycerides

Treatment with *Hibiscus tiliaceus* leaf extract at both doses of 250 mg and 500 mg resulted in a significant increase in serum HDL levels compared to the induction group. The serum cholesterol and triglyceride levels were also significantly decreased compared to the induction group, as shown in Tables 2, 3 and 4. The corresponding results are presented in Figures 2, 3 and 4.

**Table 2:** Effect of treatment on serum HDL

Groups	HDL
Induction	33.94±2.870
250 mg	44.72±7.492^*^
500 mg	73.41±7.667^*^
250 mg+Xenical	67.84±5.828^*^
Xenical	70.70±5.984^*^

Data are presented as mean±standard deviation (*) significant difference (p<0.05) when compared to the induction group

**Table 3:** Effect of treatment on serum cholesterol

Groups	Cholesterol
Induction	233.97±8.498
250 mg	207.85±2.918^*^
500 mg	190.36±6.289^*^
250 mg+Xenical	188.33±7.731^*^
Xenical	191.28±8.262^*^

Data are presented as mean±standard deviation (*) significant difference (p<0.05) when compared to the induction group

**Table 4:** Effect of treatment on serum triglycerides

Groups	Triglycerides
Induction	219.18±11.608
250 mg	186.81±6.969^*^
500 mg	162.47±1.787^*^
250 mg+Xenical	166.92±3.877^*^
Xenical	164.85±4.143^*^

Data are presented as mean±standard deviation (*) significant difference (p<0.05) when compared to the induction group

### Effect of Xenical on lipid profile

Treatment with Xenical leaf extract (both doses 250 mg and 500 mg) resulted in a significant increase in serum HDL and decreased serum cholesterol levels and triglyceride levels compared to the induction group, as shown in Tables 2-4 and Figures 2-4, respectively.

### Effect of combining herbal extract and Xenical on lipid profile

Treatment with a combination of herbal extract and Xenical significantly increased serum HDL levels and decreased serum cholesterol and triglyceride levels compared to the induction group, as shown in Tables 2-4 and Figures 2-4, respectively.

## DISCUSSION

In this study, we demonstrated that repeated administration of *Hibiscus tiliaceus* leaf extract at doses of 250 mg and 500 mg, as well as Xenical alone and in combination with the leaf extract over an 8-week period, exhibited anti-obesity effects compared to the induction group.

Our data indicate that the combination of *Hibiscus tiliaceus* leaf extract (250 mg) with Xenical (Group IV) had the most pronounced anti-obesity effect. The exact mechanism by which this herbal extract exerts its anti-obesity effect remains unknown. However, since obesity has been associated with increased oxidative stress [27], it is plausible that the anti-obesity effect of *Hibiscus tiliaceus* is attributed to its antioxidant properties [28].

Repeated administration of both doses of *Hibiscus tiliaceus* leaf extract alone, Xenical alone, and their combination over 8 weeks resulted in a significant decrease in cholesterol and triglyceride levels compared to the induction group. Additionally, a significant increase in HDL (high-density lipoprotein) levels was observed compared to the induction group.

Our data demonstrate that treatment with *Hibiscus tiliaceus* leaf extract (500 mg) (Group II) was the most effective in reducing triglyceride levels compared to the induction group, while Xenical (Group V) showed the greatest efficacy in increasing HDL levels compared to the induction group. Furthermore, the combination of 250 mg of the leaf extract with Xenical (Group IV) was the most effective in achieving these outcomes.

The exact mechanism by which the leaf extract affects the lipid profile remains unknown. However, the observed effects may be due to decreased cholesterol synthesis and fatty acid production [29]. Xenical functions by reducing the intestinal absorption of saturated fat, thereby lowering serum cholesterol levels [30].

Study limitations include the relatively small sample size; therefore, more research is needed to confirm the results. Additionally, the long-term effects of the *Hibiscus tiliaceus* leaves extract and orlistat combination remain unclear. The findings should be interpreted with caution since more research is needed to fully understand the potential of *Hibiscus tiliaceus* leaves extract in treating obesity.

## CONCLUSION

In conclusion, the study suggests that *Hibiscus tiliaceus* leaves extract alone and in combination with orlistat can effectively reduce body weight and fat mass, improve lipid profile, and promote sustainable agricultural practices. However, the study's findings should be interpreted with caution since the study was conducted on rats, and more research is needed to confirm the effectiveness of the extract in treating obesity in humans. The use of synthetic drugs like orlistat can have harmful side effects, and more research is needed to determine the relative contribution of the extract and orlistat to the observed effects.
